# *Ad Hominem* Criminalisation and the Rule of Law: The Egalitarian Case against Knife Crime Prevention Orders

**DOI:** 10.1093/ojls/gqab041

**Published:** 2021-12-28

**Authors:** Alex Green, Jennifer Hendry

**Affiliations:** 1 Lecturer in Law, York Law School, University of York; Academic Associate, 23 Essex Street Chambers; 2 Associate Professor of Law and Social Justice, University of Leeds School of Law; Academic Associate, 23 Essex Street Chambers

**Keywords:** *ad hominem* criminalisation, Rule of Law, legal theory, knife crime, regulation

## Abstract

This article advances a novel account of ad hominem criminalisation that draws upon a distinct theory of the Rule of Law and its egalitarian foundations. Employing the recent and controversial example of Knife Crime Prevention Orders, as established by the Offensive Weapons Act 2019, it argues that the concept of civic equality is central to understanding the vice of ad hominem criminalisation as an aberrant form of government by law. This vice consists in the manner that such criminalisation individualises, differentiates and instrumentalises the regulatory subject, placing them outwith the bounds of civic equality as established by the Rule of Law.

## 1. Introduction

This article advances a novel theory of *ad hominem* criminalisation, which builds upon the typical definition of that phenomenon as the creation of new criminal offences targeted at distinct individuals or groups, rather than at society as a whole.^1^ It makes two primary contributions: first, it argues that *ad hominem* criminal legislation undermines government by law as a distinct type of political association by violating the ‘civic equality’ that the Rule of Law exists to protect. Second—and this may run contrary to the intuitions of some lawyers—it contends that many of the most insidious instances of *ad hominem* criminalisation are *implicit*, appearing in legislation that, on its face, appears sufficiently general to be Rule of Law compliant. These two insights matter not only because they represent significant advances within the legal theory, but also because they help diagnose important social injustices. To demonstrate this point, and to provide our account of *ad hominem* criminalisation with concrete illustration, we examine sections 14 and 21 of the Offensive Weapons Act 2019, which empower courts to impose Knife Crime Prevention Orders (KPCOs) on individuals without the need for criminal conviction. Notwithstanding their general language, we argue that these provisions create an *ad hominem* criminal regime when read, as they must be, in light of the social context within which they are to operate.

Standard accounts of *ad hominem* criminalisation characteristically present that phenomenon in terms of explicit discrimination: *ad hominem* legislation singles out, in a manner that is plain to see, some individual or group who is to receive differential treatment under the criminal law.^2^ The classic illustration of this position is *Kable v Director of Public Prosecutions (NSW)*.^3^ In that case, the High Court of Australia upheld Kable’s challenge to the legality of an order made against him in compliance with the Community Protection Act 1994, enacted by the Parliament of New South Wales. The 1994 Act targeted Kable by name (sections 3 and 5) and, in effect, compelled the Courts of New South Wales to make a *sui generis* detention order against him. As convincingly argued by Allan, the rationale for the decision in *Kable* must be understood in terms of the separation of powers: ‘the separation of judicial power and fundamental status of the ordinary judicial process, as precepts intrinsic to the rule of law, is inconsistent with unfettered power of arbitrary action [by] the legislature’.^4^ Furthermore, the importance of the separation of powers in this context lies downstream from the duties that courts have to ‘uphold the dignity and independence of the citizen’.^5^

Whilst we agree with Allan’s analysis of *Kable* as a commonly accepted instance of *ad hominem* criminalisation, we do not believe he goes far enough. According to him, ‘there is no insuperable objection to the making of detention orders by courts outside their ordinary function of sentencing convicted offenders … [provided that] such detention [is authorised] in general terms, in principle applicable to all’.^6^ Our objection is that even legislation couched in general terms may, through the *implicit* targeting of particular individuals or groups, undermine the civic equality that grounds the Rule of Law. It is for this reason that our more expansive account of *ad hominem* criminalisation has been developed.

Our argument begins by articulating the Rule of Law’s egalitarian foundations, upon which our theory is constructed (section 2). Having done so, we present our definition of *ad hominem* criminalisation, which conceptualises this problematic form of regulation as one that, through the use of individualised control, either explicitly or implicitly differentiates between the status of (i) particular individuals or groups and (ii) society at large, in such a way as to instrumentalise the treatment of the former for the benefit of the latter (section 3). What may seem counterintuitive, but nonetheless supplies our approach with analytical potency, is the manner in which we say that the relevant implication should be ascertained. Rather than relying only upon the text of the relevant statute(s), or even that text in combination with considerations of general principle, we contend that close attention must be paid to the broader sociolegal context within which the relevant legislation either is or is likely to be applied. We illustrate this distinct approach through a critical analysis of the KCPO provisions in the 2019 Act, which we say, notwithstanding their seemingly general form, perfectly exemplify the distinct combination of individualisation, differentiation and instrumentalisation that constitutes *ad hominem* criminalisation (section 4). As we shall see, one radical implication of this approach is that the category ‘*ad hominem* criminalisation’ is far broader than has been traditionally conceived.

## 2. The Rule of Law and Civic Equality

Following Allan (and others), we contend that the most instructive way to characterise the distinct wrongfulness of *ad hominem* criminalisation is to attend the ways in which it violates the Rule of Law. As such, the natural place to begin our analysis is with what we take the latter concept to entail. The Rule of Law is a complex and essentially contested concept.^7^ In what follows, we outline a distinct account of its egalitarian foundations that captures the idea that communities who govern themselves in accordance with law thereby institute ‘civic equality’. To explain the conception of civic equality we have in mind, this section will proceed as follows. First, we distinguish the ‘vertical dimension’ of the Rule of Law, which concerns the relationship between citizens and the state, from its ‘horizontal dimension’, which concerns the relationship *between* citizens. Second, we argue that the Rule of Law’s horizontal dimension is concerned with the *equal status* of citizens as legal subjects. Third, we further delineate such status-based equality by contrasting it with other (connected but distinct) egalitarian considerations, which we examine through the notion of *un*equal laws. Fourth and finally, we detail the unique (and morally complex) place that contemporary criminal justice holds within a theory of the Rule of Law conceived in these substantively egalitarian terms.

### A. The Two Dimensions of the Rule of Law

The Rule of Law has both horizontal and vertical dimensions.^8^ Vertically, it regulates the relationship between citizens and the state, requiring that ‘force not be used or withheld, no matter how useful that would be to ends in view, no matter how beneficial or noble those ends, except as licensed and required by … past political decisions about when collective force is justified’.^9^ This emphasis upon legality as ‘the enterprise of subjecting human conduct to the governance of rules’ represents perhaps the most generally accepted aspect of the Rule of Law.^10^ It not only appears in the work of Dworkin and Fuller (just quoted), but also in that of Finnis,^11^ Hayek,^12^ MacCormick,^13^ Raz^14^ and Waldron,^15^ to list but a few. According to this tradition, law functions as a limitation upon *arbitrary* power, in that it forbids fully discretionary decision making by government officials.^16^ In what follows, we build upon this insight, although we remain agnostic as to whether the vertical restrictions that the Rule of Law imposes are purely formal and procedural, or whether they also include more substantive protections.^17^ As a minimum, we take these restrictions to include Fuller’s eight principles of legality, which hold that laws must be: (1) sufficiently general; (2) publicly promulgated; (3) prospective; (4) sufficiently intelligible; (5) non-contradictory; (6) relatively constant; (7) possible to obey; and (8) administered consistently with their apparent meaning.^18^ Following Raz, we accept that these vertical safeguards not only restrict arbitrary decision making, but also enable personal autonomy by facilitating individual planning.^19^ Nonetheless, what interests us here is not so much autonomy as equality and the ways in which the latter concept can be instantiated by law.

This brings us to the Rule of Law’s horizontal dimension, which concerns the particular ‘mode of association’ that government establishes by law within a political community.^20^ We expand upon the egalitarian nature of this association in the following subsection, but it might be briefly stated as follows: by living within communities regulated in accordance with a common set of standards, individuals within those communities are treated as fundamentally *equal in status* to those they live alongside.^21^ The horizontal dimension of the Rule of Law secures this equality by requiring of *everyone* an attitude that has sometimes been called ‘fidelity’: one of respect, which recognises and values the fact that *this* community is a community of laws.^22^ As defined by Postema, fidelity entails that everyone—that is, not just public officials, but also private individuals—takes an active role in holding each other to account under those same standards that, vertically speaking, constrain governmental power.^23^ Such a community is one in which a people have defined themselves, at least in part, in terms of their mutual and reciprocal adherence to *one* set of governing standards. They have, to put matters in Arendtian parlance, ‘produced equality by organisation’, even though it be equality of a very particular and limited kind.^24^ Our concern, which we expand upon in what follows, is that certain kinds of governmental activity can undermine the status-based equality instantiated by the horizontal dimension of the Rule of Law and so violate the social contract implicit in this valuable mode of association.^25^ In this article, our chief concern is *ad hominem* criminalisation—exemplified (for us) by the Offensive Weapons Act 2019 and its creation of KCPOs—as a practice deeply at odds with the kind of civic equality that underpins government by law.^26^

### B. Communities of Law and Civic Equality

In this subsection, we detail the Rule of Law’s egalitarian foundations. As noted above, our core claim at this stage is that a community with the Rule of Law is one in which a particular kind of status-based equality has been instituted. The status in question is one of *civic equality* and turns on mutual recognition, both of personhood and of a shared identity as part of ‘the people’ who collectively constitute the relevant political community.^27^ Such recognition picks out the individuals concerned, to paraphrase Nagel, as occupying a certain sort of ‘place’ within that community’s normative landscape.^28^

Civic equality, which for present purposes we define as equal subjection to a single set of governing principles, need not be particularly ‘thick’ and may well admit, for example, significant socio-economic inequality. However, it does require that there be no ‘thumb on the scale’ when it comes to how particular individuals should be treated, or how they are expected to behave.^29^ To be fully respected as equal members of a community constituted by law, each citizen must be both protected by and held accountable to the same set of governing principles.^30^ In horizontal terms, this requires that all citizens demonstrate ‘faithfulness … to each other with respect to some common governing norms’.^31^ Any individual who violates those common standards without appropriate justification will implicitly disrespect and, in extreme cases, actively undermine civic equality.^32^

Claims of this kind, while perhaps less widespread in contemporary philosophy than those dealing with the vertical dimension of the Rule of Law, have considerable pedigree. Rousseau, for example, famously argued that laws properly so called—which is to say, those created in compliance with popular sovereignty—must address the public *as a whole*, rather than particular individuals or subgroups.^33^ On this view, any legislation that is not ‘general’ cannot truly be said to govern a community of collectively sovereign equals.^34^ Indeed, Rousseau went so far as to suggest that such partial directives cannot really be considered law at all.^35^ Although we make no commitments as far as this more extreme claim is concerned, we accept a broadly Rousseauian view of the relationship between civic equality and the Rule of Law. Specifically, we contend that any directive that picks out particular individuals or subgroups as fundamentally different *vis-à-vis* their basic status as citizens, and thereby denies their civic equality, is at odds with the values that underpin the horizontal dimension of the Rule of Law.^36^ As such, laws of this kind constitute aberrant examples of law as an ideal type, even though they might be sound *qua* law in all other respects: that is, not only legally valid, but also clear, prospective, stable and so on.^37^ In this respect, we are in fundamental disagreement with theorists such as Fuller, who would consider this kind of substantive generality to be an ‘external’ moral issue and not part of the ‘inner morality of law’.^38^

The argument that anchors the Rule of Law to civic equality is this: whilst there might be many reasons to institute and maintain a legal system—the avoidance of coordination problems,^39^ the maintenance of civil peace^40^ and so on—one important reason is the institutionalisation of equal treatment through uniform standards of behaviour.^41^ As Dworkin notes,^42^ government by law ‘fuses citizens’ moral and political lives: it asks the good citizen, deciding how to treat his neighbor when their interests conflict, to interpret the common scheme of justice to which they are both committed just in virtue of citizenship’.^43^

This is practically advantageous in circumstances where people disagree about what values such as justice, fairness and equality truly require.^44^ Although no single set of legal standards will cohere with every divergent view, any set that is sufficiently general will establish each citizen as equally subject to the same governing principles, such that they need not fear becoming subject to the purely personal convictions of anyone else.^45^ This permits all citizens to identify with the law that governs them, albeit in an attenuated manner. For, although such laws may not ‘belong’ to any one person, in that those directives did not originate from purely personal acts of will, the very impersonality of law means that properly constituted legal standards belong to one individual *no less* than they do to any other.^46^ Indeed, under such conditions, intentional law-breaking can be interpreted as the substitution of these impersonal standards for personal judgment. Absent appropriate justification, such substitution risks the implicit assertion that compliance with one’s own desires or beliefs should trump the maintenance of civic equality. Such behaviour disrespects the distinct and valuable mode of association the Rule of Law represents. This, we contend, is the true normative weight and underlying value behind Dicey’s famous requirement that ‘every man, whatever his rank or condition, is subject to the ordinary law of the realm and amenable to the jurisdiction of ordinary tribunals’.^47^

### C. The Problem of Unequal Laws

That some laws (whether criminal or otherwise) can promote *in*equality poses no serious objection to the position outlined above. Whilst there is nothing about governance by general principles that necessarily excludes standards with inegalitarian consequences,^48^ it does not follow that this mode of association lacks all ‘equality-value’. To see why, an analogy is helpful. It is generally accepted that our personal autonomy is served by us making choices for ourselves. However, it also seems clear that some choices may limit our overall and ongoing autonomy. For example, remortgaging one’s home to buy a Tesla would likely have injurious financial consequences that would seriously limit the autonomy of the individual in question. Nonetheless, it would be both eccentric and unmotivated to claim that such unwise choices had *no* value as far as our personal autonomy was concerned. If they are truly *free* choices, in the sense of being both unforced and uncoerced,^49^ then they are also instances of that autonomy being exercised. The interesting point in relation to such cases seems to be that their ‘autonomy-value’ is qualified. They both instantiate and frustrate autonomy: exemplifying it as a means whilst producing a counterproductive end. This may make them qualified examples of autonomous decision making, but they remain examples nonetheless.

The position of inegalitarian laws is similar. On the assumption that such principles are sufficiently general, in that they speak to the public as a whole rather than to status-differentiated individuals or subgroups, the fact that they may produce inegalitarian outcomes does not altogether negate their equality-value as a means for governance. For example, laws relating to inheritance and bequeathment arguably play a significant role in the perpetuation of social injustice by maintaining inequality of resources.^50^ Nonetheless, they are sufficiently general in character to govern entire political communities and so evince no implicit denial of civic equality *as an individual status*. Like free choices that limit our long-term autonomy, inegalitarian laws occupy a complex position: they instantiate equality as a means whilst undermining it as an end. They are, to this extent, qualified examples of government by law whilst nonetheless remaining meaningful institutionalisations of civic equality.

Note that to accept the qualified equality-value possessed by laws with inegalitarian consequences is not to endorse what is sometimes called ‘formal’ equality. That principle, given its clearest expression by Aristotle, stands merely for the logical precept that ‘like cases must be treated alike’.^51^ Exclusive reliance upon it within legal reasoning has often been critiqued as little more than disguised prejudice and nothing about our position seeks to prove otherwise.^52^ Instead, we take substantive equality to be a multifaceted concept of which status-based civic equality represents just one dimension.^53^ Civic equality of the sort that interests us stands in particularly strong relation to the Rule of Law; however, it is not the only form of substantive equality that law can instantiate. Good laws will often promote social, economic or religious equality, to name but a few examples. Some of these egalitarian advances may touch upon civic status, whilst others will not. Concurrently, laws with inegalitarian consequences remain bad laws to that extent, even though they may nonetheless institutionalise *civic* equality. They may, for example, produce unequal distributions of wealth, opportunity or welfare.^54^ But this alone cannot entail status-inequality: deeper differentiation of that kind will only be present to the extent that divergent conceptions of the legal subject *qua* citizen (or otherwise) can be located within the law.^55^ As we argue below, *ad hominem* criminalisation, exemplified by the KCPO, is paradigmatically status-inegalitarian in this way.

The conceptual advantages of the view articulated in this subsection are threefold. First, our analysis maintains the important distinction between *laws which are bad*—because, say, they produce inegalitarian outcomes—and *bad laws*: those lacking the characteristic virtues the Rule of Law promotes. Second, by nonetheless emphasising the connection between government by law and the institutionalisation of equality, it draws attention to the conceptual tensions created by laws that institute *civic* equality whilst simultaneously undermining adjacent egalitarian values. Third, and most importantly for present purposes, it clarifies a distinct way in which *particular* laws undermine the Rule of Law. As we emphasise below, any law that differentiates between (i) the civic status of certain individuals or subgroups and (ii) the status enjoyed by the public at large cannot comply with civic equality.^56^ This sets the law in question directly at odds with the distinct mode of association instituted by the horizontal dimension of the Rule of Law.

### *D*. Civic Exclusion and the Distinctiveness of *Ad Hominem* Criminalisation

This complex relationship between the Rule of Law and (civic) equality pertains, we argue, in respect of *all* laws. Within this broader rubric, however, criminal laws hold a special and problematic place. This pertains due to the latter’s characteristic use of *punishment*, which, as Duff explains, ‘express[es] the polity’s condemnation of the offender’s conduct as a wrong’, such that the substantive prohibitions of criminal law ‘define wrongs that merit the condemnation and punishment for which [criminal justice as a whole] provides’.^57^ Such punishments consist in various restrictions upon liberty, some more extreme than others. Most importantly, many criminal punishments restrict the *physical* liberty of those subject to them, whether through curfew, geographical limitations upon free movement or incarceration. Criminal punishment in this mode contrasts starkly with the core functions of private law, for example, which are to compensate and to offer restitution.^58^

Although he deplores this feature of contemporary criminal justice, Duff accepts that punishment of the above sort often operates so as to ‘treat those who violate our criminal laws as outsiders who have forfeited their status as citizens’.^59^ By limiting the physical freedom of convicted offenders, criminal law does more than express the fact that they committed a wrong, which private law arguably also accomplishes by publicly decreeing someone liable to compensate someone else.^60^ Insofar as they impose limitations upon physical liberty—and thereby *factually* exclude individuals from full social participation—such characteristic criminal punishments *expressively* exclude subject individuals from full civic equality, at least while their factual exclusion continues to pertain.^61^ It is this exclusionary function—this demarcation of civic ‘outsiders’^62^—that we take to be the most distinctive and problematic aspect of criminal regulation.^63^ Whereas private law may express censure for various wrongs committed by individuals *within* the same polity, criminal law demarcates particular conduct as *so* wrongful that it (at least temporarily) disrupts the status of the perpetrator as a member of society with full civic privileges.^64^

Given the above, the close connection between the Rule of Law and civic equality poses a radical possibility: namely, that, at least on its face, punitive criminal justice *qua* exclusionary technique seems incompatible with the egalitarian basis of legality *in general*. This is so because the essence of civic equality is that of (status-based) inclusion, whilst the *sine qua non* of criminal justice is that of exclusion. Grappling with that larger issue falls outwith the scope of this article, although the prospect remains intriguing. For present purposes, we acknowledge two possibilities: first, that some appropriate argument might show how punitive social exclusion could be rendered normatively compatible with civic equality, such that criminal justice might be ultimately justifiable *vis-à-vis* the Rule of Law; and second, that no such compatibility is possible, such that punitive regulation (and therefore criminal law as such) must ultimately be rejected as inconsistent with legality. The point for now is that whichever alternative proves true, our account of *ad hominem* criminalisation remains both practically important and theoretically interesting. This is so because even if criminal punishment violates civic equality in general, *ad hominem* criminalisation, as we argue, constitutes a uniquely complex and egregious violation of that sort. Moreover, by explaining *why*, we provide a point of departure for more general critiques of criminal justice from a status-egalitarian point of view. Conversely, should some convincing integration of criminal justice and egalitarian legality become available, our arguments would survive by way of demonstrating that *ad hominem* criminalisation necessarily falls *outwith* any such integrated justification.

Ultimately, both eventualities turn on the same substantive proposition, upon which our argument elaborates at length: that *no* set of criminal prohibitions can be justified *vis-à-vis* status-egalitarianism unless *all* members of the relevant polity are governed by that set and *only* that set. Any divergence from this, whether in the form of personal exemption from or asymmetric subjection to additional criminal regulation, does significant expressive harm civic equality.^65^ This is so because: (i) the very status of civic equals is instantiated and expressed by their subjection to the same scheme of governing principles; and (ii) the onerous and exclusionary nature of criminal regulation heightens the need for justification in these terms. To hold anyone personally exempt from regulation under the full set of criminal laws that apply to their compatriots does expressive harm to the horizontal equality that the Rule of Law maintains. Moreover, and crucially for our arguments below, to single out any individual or group for *additional* criminal regulation harms civic equality in precisely the same manner and, under certain circumstances, to a far greater degree. Therefore, whatever the general compatibility between criminal justice and the Rule of Law may be, *ad hominem* criminalisation, as we define that phenomenon, remains manifestly impermissible.

## 3. *Ad Hominem* Criminalisation and the Rule of Law

In this section, we argue that *ad hominem* criminalisation constitutes a distinctively egregious violation of civic equality. To do so, we begin by returning to the distinction between the horizontal and vertical dimensions of the Rule of Law, arguing that some governmental wrongdoing under the vertical dimension of that concept is best characterised as an intentional or careless violation of the civic equality that its horizontal dimension exists to protect. Next, we contend that *ad hominem* criminalisation represents a paradigmatic mode of such governmental wrongdoing and, moreover, one with a unique structure. Namely, we say that *ad hominem* criminalisation occurs whenever ‘individualised’ criminal regulation, either explicitly or implicitly, differentiates between the status of (i) particular individuals or groups and (ii) society at large in such a way as to instrumentalise the treatment of the former for the benefit of the latter. It is the distinct combination of these three elements—individualisation, differentiation and instrumentalisation—that renders *ad hominem* criminalisation so uniquely egregious.

### A. Horizontal Violation through Vertical Breach

The previous section distinguished two dimensions of the Rule of Law to elucidate the connection between that concept and the value of civic equality. In this subsection we draw an important connection between ‘horizontal’ and ‘vertical’ legality by arguing that certain kinds of governmental wrongdoing are best characterised as attacks upon the distinct ‘mode of association’ that the Rule of Law creates. In the first place, as Dworkin’s maintains, government according to law matters

not merely in the sense that the law is enforced as written, but in the more consequential sense that government must govern under a set of principles in principle applicable to all. *Arbitrary coercion or punishment violates that crucial dimension of political equality, even if, from time to time, it does make government more efficient*.^66^

Arbitrary governmental decision making violates civic equality for similar reasons to individual law breaking. Whether operating as group agents or as collections of individuals,^67^ governments acting outside their legal authority substitute the behaviour law requires with a freestanding and unlicensed decisional act, in much the same way that an individual citizen might. This disrespects civic equality because it casts governments as more than the executive agents of their people, constituted for the purposes of securing their communities of law. Rather, it implies them to be rulers in the more elevated sense of social or civic superiors, justified in issuing unilateral decrees to their inferior subjects.^68^ This abrogation of equal status is contrary to the horizontal dimension of the Rule of Law even though it manifests as a *vertical* breach of the social contract between the rulers and the ruled.

Indeed, such vertical violations arguably attack the egalitarian spirit of the Rule of Law more vociferously than violations on the part of any individual citizen. As Murphy argues, all obligations to obey the law, even though grounded in the same basic set of moral considerations, are agent-relative in that their normative weight depends upon the harm non-compliance would do to government under law itself.^69^ Governments are uniquely placed to do considerable harm in this regard, given: (i) their pivotal role in securing the Rule of Law within our public institutions; and (ii) their relative potency *vis-à-vis* the personal power of most private individuals. Violations of legality perpetrated by governments also have significant *expressive* impact because of the trust that is necessarily placed in them by their people.^70^ Within a community of law, such acts subvert a core purpose of government: the maintenance of civic equality through the institution of law.

In what follows, we argue that arbitrary coercion or punishment is not the only form of harm that our rulers can perpetrate against the Rule of Law: it can also be attacked through acts that take legal form but nonetheless run contrary to civic equality. Specifically, we contend that any laws engaged in *ad hominem* criminalisation are incompatible with the status-egalitarian foundations of legality, even if they are otherwise Rule of Law compliant. Such *ad hominem* laws go beyond creating inegalitarian *outcomes*, such as creating or maintaining unjustifiable resource distributions. Instead, they either explicitly or implicitly target particular individuals or subgroups, differentiate them as ‘enemies’ or ‘dangerous others’^71^ and then instrumentalise them as a means for the security of the majority.^72^ This undermines the status *as citizens* of those affected by placing them in a tripartite, asymmetric and ultimately unjustifiable regime of criminal regulation.

### B. *Ad Hominem* Criminalisation and Civic Equality

‘The most fundamental objection’ to instances of *ad hominem* legislation ‘is their violation of the principle of equality that there should not be “a special rule for a particular person or a particular case”’.^73^ This stems, as Allan explains, from the more general proposition:

Victimization of unpopular persons or despised minorities is excluded not merely by the requirement that coercion must be authorized by general rules, but by the further requirement that those rules should themselves be justified by policies or principles consistent with the ideal of equal citizenship.^74^

In section 2, we described this ideal of civic equality in terms of the distinct ‘mode of association’ established by the Rule of Law’s horizontal dimension and, in the previous subsection, we further argued that particular kinds of governmental wrongdoing can undermine these (status-based) associative bonds. In this subsection, we characterise *ad hominem* criminalisation as one distinctive mode of such wrongdoing, building upon the insights of Allan and others to offer a general definition that captures its uniquely egregious violation of civic equality. On our account, *ad hominem* criminalisation exists wherever law, through the use of individualised control, either explicitly or implicitly, differentiates between the status of (i) particular individuals or groups and (ii) society at large in such a way as to instrumentalise the treatment of the former for the benefit of the latter. These three elements—individualised control, differentiation and instrumentalisation—each raise distinct moral issues *vis-à-vis* status-egalitarianism. Moreover, when *all* are instantiated within the *same* legal directive, civic equality is violated to an uniquely egregious degree.

Individualised control, to quote Crawford, consists in ‘tailoring the response and regulatory regime—the rules, obligations, conditions, support arrangements, mechanisms for enforcement and monitoring outcomes—to the circumstances of those who are the subjects of regulation’.^75^ Whatever its other merits might be, this kind of regulation creates a formal asymmetry between those subject to it and those ‘ordinary’ citizens who remain regulated by non-individualised legal standards. This is problematic because, as argued above, the very status of civic equality is instantiated and expressed by a society’s uniform subjection to *the same* scheme of governing principles. Any formal asymmetry that seemingly diverges from this mode of association condition must therefore demonstrably cohere with civic equality *in substantive terms* or stand in violation of that ideal. In the criminal context, particularly given the exclusionary nature of punitive sanctions, this entails that such asymmetry imposes a uniquely weighty burden upon government to demonstrate that individualised criminal regulation ‘corresponds to an intelligible requirement of the public good’, such that civic equality is publicly observed.^76^ Crucially, given the demands of civic equality, this public good cannot be ‘the utilitarian “greatest good of the greatest number”’ but must be that of ‘an *all-round association*’, capable of ‘securing … a whole ensemble of material and other conditions that tend to favour the realisation, by *each individual in the community*, of his or her personal development’.^77^ This means that, for individualised control to be consistent with the egalitarian foundations of the Rule of Law, the asymmetry it generates must be justified by an appeal to the security of a body politic to which the subject individual possesses equal membership (and therefore equal civic status). If no such justification is provided—or if what has been provided is normatively implausible—then the *prima facie* violation of civic equality represented by the formal asymmetry of individualised control will truly undermine civic equality. Our concern is that, in the case of genuine *ad hominem* criminalisation, the twin vices of differentiation and instrumentalisation operate so as to implicitly deny equal status of this kind. In so doing, these vices both compound and confirm the *prima facie* expression of status-inequality individualised control affects.

Differentiation, in the relevant sense, must be fundamental, speaking to the status of an individual or group *qua* member(s) of the broader community. Such basic differentiation has two indicative features. First, unlike legal categories that demarcate particular individuals as ‘special’ for more discrete purposes—as trustees, contractual parties or creditors, for example—the differentiation that grounds *ad hominem* criminalisation is non-consensual. For example, in *Liyanage v R*, legislation struck down by the Privy Council had purported to create a new criminal offence that retrospectively targeted those perceived responsible for a failed political insurrection in Sri Lanka.^78^ Perhaps obviously, this *ex ante* criminalisation did not take the form of a voluntarily adopted role but of an imposed status alteration.

The second indicative feature of the differentiation that marks *ad hominem* criminalisation, which *Liyanage* also exemplifies, is that it casts the subject individuals or subgroups as ‘outsiders’, in the sense that they are *negatively* contrasted, at the level of their political and social status, with the community at large. Most importantly, this invidious differentiation must *attach* to the presence of individualised control, such that it is in large part their being targeted by the relevant legal techniques that precipitates these individuals suffering an alteration of status.^79^ Whether explicitly or implicitly, the individualised control at issue must violate the egalitarian maxim discussed in section 2: that the whole polity must be governed by a single set of criminal laws and that if it is not—that is, if some individuals are subject to more criminal regulations than others—then civic equality, and the very cohesion of that polity as a community of equals, is undermined.

In extreme cases, such problematically differentiating laws present the individuals or subgroups they target as ‘enemies’ faced by the larger community,^80^ who not only ‘threaten’, but also thereby come to define that community through rhetorical contradistinction.^81^ As explained by Carvalho, within such differential regulation,

Anxiety … is taken to its ultimate consequences, as responsible subjects are taken to have the capacity to seriously endanger the integrity of the political community by means of their agency …any individual who is identified as potentially dangerous has to be contained before s/he can put the community at risk.^82^

This intentional division of our political communities into ‘virtuous majorities’ and ‘dangerous others’ strikes at the very heart of civic equality.^83^ It creates, in effect, two (status-based) classes of citizen: those to whom the ordinary criminal law applies, with its emphasis upon proven wrongdoing and personal responsibility, and those who are treated as ‘naturally’ suspect, whose criminality is a function of their legally and socially constructed identity, not the fact that they have engaged in criminal behaviour under the complete set of laws that apply to their compatriots.^84^ Even though they might not breach any formal desiderata of the Rule of Law, any laws that enact differentiations of this kind are necessarily incompatible with the egalitarian foundations described in section 2. Any mode of association that differentiates between the fundamental status of individuals in this prejudicial manner cannot embody the ideal of civic equality. As such, it represents an internally conflicted example of legal regulation and an aberrant case of government by law.^85^

The harm done by individualised control that differentiates in this way is further compounded by the final feature of *ad hominem* criminalisation: instrumentalisation. Within a community committed to civic equality, each individual must be deemed worthy of what Dworkin referred to as ‘equal concern and respect’.^86^ This is echoed by Finnis, as quoted above, when he speaks of the Rule of Law as requiring ‘each member of the community’ (that is, not just the majority or an ‘elite’) to be treated as meriting the opportunity for ‘personal development’.^87^ Compliance with principles of this sort need not imply thoroughgoing egalitarianism, in the sense of promoting equality of welfare, resources or capacities.^88^ However, it does necessitate that each citizen be treated as fundamentally equal in *status* to every other. The instrumentalism that marks *ad hominem* criminalisation undermines this important egalitarian maxim by setting apart subject individuals in another, more insidious manner than the problematic differentiation discussed above. By deploying individualised control in response to the perceived *danger* that certain individuals or subgroups represent, *ad hominem* criminalisation imposes additional criminal regulations upon those persons in the name of collective security.^89^ This emphasis on the security of the majority, when procured through the asymmetric treatment of those subject to differential regulation, implicitly communicates the justifiability of treating those people as a *means* to a majoritarian *end*.

Instrumentalisation does discrete expressive harm to the notion of equal status that magnifies the existing divisions communicated by individualisation and the differentiation of the body politic into ‘ordinary citizens’ and ‘dangerous others’. Any political community that regulates in this instrumental way is, to use Kantian parlance, incapable of existing as a ‘kingdom of ends’, within which each individual is recognised as being of *absolute* value, rather than simply of the *relative* value imparted by their instrumental utility towards securing some end.^90^ Given that the horizontal dimension of the Rule of Law entails a commitment to the equal status of each citizen, it cannot be reconciled with the variability that such relative value implies. As such, any purely instrumental treatment will be at odds with civic equality and will violate the egalitarian precepts of the Rule of Law as a distinct mode of association. *Ad hominem* criminalisation, insofar as it licenses the individualised control of some citizens purely as a means to promoting the security of others, violates this egalitarian maxim in precisely that way.

To summarise, *ad hominem* criminalisation represents a distinctively egregious violation of the Rule of Law. By deploying individualised control as a purely instrumental means for promoting public security, and by representing those subject to that control as *outwith* the community proper, *ad hominem* criminalisation undermines civic equality via a threefold signification that not all citizens qualify for regulation by the laws that govern more ‘virtuous’ or ‘ordinary’ people. In what remains, we argue that Knife Crime Prevention Orders, as established by sections 14 and 21 of the Offensive Weapons Act 2019, are paradigmatic of this problematic regulatory approach and, as a result, that they stand in an uneasy relationship with the Rule of Law.

## 4. Knife Crime Prevention Orders as an *Ad Hominem* Regime

The Knife Crime Prevention Order was introduced with the stated aims of: (i) reducing the rising rates of knife-related violence in England and Wales; and (ii) equipping police with the means to ‘divert those who may be carrying knives … away from being involved in knife crime’.^91^ A hybrid civil/criminal preventive procedure, KCPOs are ostensibly civil in character but carry criminal sanctions in the event of breach.^92^ In this regard, they mirror the two-step structure of the controversial and now-defunct Antisocial Behaviour Order (ASBO)^93^ and other civil preventive orders.^94^ We argue that the escalatory and hybrid structure of KCPOs, together with the sociolegal context that is likely to occasion their application and enforcement, places them in fundamental tension with the Rule of Law.^95^

Specifically, we contend that sections 14 and 21 of the 2019 Act create an *ad hominem* criminal regime—targeting a young, urban and predominantly minority ethnic population—notwithstanding the general manner in which those provisions are phrased. First, we outline the legal framework that makes KCPOs available in the absence of a criminal conviction.^96^ Second, we detail the ways in which KCPOs constitute individualised criminal regulation. Third, we provide an argument in favour of the notion that the 2019 Act problematically differentiates between the intended targets of KCPOs and the remainder of society. Finally, we claim that this differentiation is motivated by a logic of securitisation, which wrongfully instrumentalises the set of individuals envisioned as becoming subject to KCPOs. Thus, all three elements of *ad hominem* criminalisation are made out.

### A. The Legal Framework

Section 14 of Part 2 of the Offensive Weapons Act 2019 provides that courts can make a KCPO in respect of a person aged 12 or over if that court is satisfied, on the balance of probabilities, that this person has ‘had a bladed article with them without good reason or lawful authority’ in a public place, school or college on at least two occasions within the preceding two years. In addition, the court must find that such an order is necessary to protect the public, or specific members of the public, from the risk of harm involving a bladed article, or to prevent the defendant from committing a crime with said bladed article. Importantly, although such an order can be made *without* a criminal conviction, significant penalties can follow. Breach of a KCPO constitutes an offence, for which punitive sanctions, such as a custodial sentence of up to 24 months, can be imposed.^97^

Individuals made subject to a KCPO are obliged to comply with specific positive and negative requirements. Under section 21 of the Act, negative requirements can comprise geographical, associational and temporal restrictions (curfew), both online and offline, while positive requirements can include duration-stipulated attendance at counselling and/or educational courses. We argue that these requirements, which ‘combine to comprise severe and lengthy restrictions upon [an] individual’s movements and activity, both online and offline’,^98^ effectively take the form of personalised laws.^99^ This is problematic in itself, rendering the individual restricted under the order subject to an asymmetric regime of regulation and control. Furthermore, it runs directly contrary to several fundamental principles of classical criminal law, notably the presumption of innocence, as well as untying the otherwise necessary correlation between criminal behaviour and criminal responsibility.^100^

### B. Asymmetry and Individualised Control

Notable here are the different values at the heart of the respective regimes of criminal justice and so-called ‘responsive regulation’. While ‘criminal justice is founded on principles of independence and impartiality … regulation promotes pluralistic and flexible pragmatism’.^101^ The effect of the move from traditional criminal law to the more dynamic options afforded by responsive regulation is, therefore, a shift from an established and publicly promulgated general standard into differential, *individualised* treatment.^102^ The positive and negative requirements of the KCPO are paradigmatic in this regard: customised behavioural controls and obligations facilitated by the order’s use of state coercion over a person who has neither been convicted of a crime, *nor has in fact committed a crime for which another member of the public would be held similarly liable*.^103^

This situation is generated, by and large, by the two-step structure of the KCPO.^104^ The undesirable behaviour is first identified by the court, which imposes requirements geared towards the prevention of said behaviour and accompanies these with notice that punishment will follow on violation of these requirements. It is in this manner that the regulation is *responsive*: non-compliance with the order’s provisions gives rise to an escalation of sanctions, with the rationale being that this operates as a disincentive to proceed further through the increasingly punitive stages of the ‘regulatory pyramid’.^105^

The procedural structure of the KCPO, as Hendry argues, thus elides the ‘classic distinction between compliance and punishment’.^106^ Consequently, an individual made subject to such an order is fast-tracked into becoming punishable by the criminal law *without* undertaking criminal activity. To be specific, *any* breach of the order can render the relevant individual liable to punishment, ranging from a custodial sentence (lasting up to 24 months) or, according to Home Office Guidance, any of the following: (i) unpaid work (Community Payback); (ii) rehabilitation activities; (iii) undertaking particular programme(s) geared towards behavioural changes; (iv) mental health treatment (with the defendant’s consent); (v) drug rehabilitation and/or a drug testing order (with consent); (vi) drug and/or alcohol treatment (with consent); and (vii) alcohol abstinence and monitoring (again, with consent).^107^ This places individuals subject to KCPOs in an asymmetric position *vis-à-vis* other members of the public, since anyone *not* subject to an order of this kind could undertake the prohibited behaviour without risking punishment. For such asymmetry to be consistent with the basic equality of citizens, the implementation of KCPOs must be capable of justification with reference to the common good. If it is not, then it violates the status-egalitarian principle that civic equality requires all legal subjects to be governed by just one set of (criminal) laws. In what remains of this article, we argue that no such justification would be plausible. The legislative intent and broader social context behind the Offensive Weapons Act 2019 render its generality purely nominal. Sections 14 and 21 create an *ad hominem* criminal regime that singles out specific populations within England and Wales, and expressively alienates them, *vis-à-vis* their basic status, from their fellow citizens.

### C. Knife Crime Prevention Orders and ‘The Dangerous Other’

As argued in section 3, *ad hominem* criminalisation can be distinguished from otherwise justifiable instances of individualised criminal regulation by the presence of morally problematic differentiation and instrumentalisation. In terms of differentiation, the starting point for analysis in the case of KCPOs must be the social context of the contemporary England and Wales and, in particular, its racial dynamics. It is in that context that sections 14 and 21 of the 2019 Act stand to be applied and, therefore, it is in light of that context that their compliance with civic equality must be judged.

In the House of Lords debate on the amendments to the 2019 Bill, Baroness Williams of Trafford explained that ‘The orders are aimed at three groups of people: young people who have been carrying a knife; habitual knife carriers of any age; and those who have been convicted of violent offences involving knives’.^108^ However, as Hendry argues in relation to these three categories, ‘without needing to be told, without needing proof, the public already believe that in the context of knife crime the offenders are young men of colour, benefit dependent, and involved in drugs, other anti-social behaviour, and low-level criminality’.^109^ These concerns were shared by more than one member of the House of Lords during that same debate. As Lord Paddick—who, prior to his retirement, served as Deputy Assistant Commissioner in London’s Metropolitan Police Service—stated:

We know the disproportionate impact that this type of order has because of our experiences with ASBOs. Stop and search is still used disproportionately on black and minority-ethnic young people, and there is no evidence to suggest that these orders will not be used in a similar way. A Youth Justice Board evaluation of ASBOs in 2006 found that 22% of young people given ASBOs were BAME—two and a half times their proportion of the population.^110^

In this sense, the general language of the Offensive Weapons Act belies the truth: that sections 14 and 21 in fact *target* young, poor, urban and disproportionately Black and minority ethnic populations. In so doing, it differentiates them in status, implicitly casting them as a danger to everybody else. The existence of this intention is once again evinced by the concerns of Lord Paddick, who notes that ‘the Government propose measures that turn youth workers into law enforcers as the supervisors of KCPOs and reinforce the belief that even the community police officers are there to arrest rather than protect you’.^111^

The true construction of any statutory provision is contingent not only upon its linguistic content and legal context, but also upon broader sociolegal considerations: upon the social background conditions within which the words of that provision operate.^112^ In the case of KCPOs, the material background conditions, as Lord Paddick’s concerns exemplify, are that England and Wales have a problem of both race and class when it comes to criminal justice. This problem is evidenced, for example, by the statistics relating to stop and search. As the Standing Committee for Youth Justice has noted, ‘black people are 40 times more likely to be stopped and searched’.^113^ This raises particular problems in the context of KCPOs because

weapons are generally hidden about the person [and so] … are more likely to be found via these methods. Black children are therefore more likely to be caught and prosecuted for possession, whether or not they are more likely than their white counterparts to be carrying a weapon.^114^

Against this background, the generality of the KCPO provisions flatters to deceive. Sections 14 and 21 might be *phrased* in general terms, but their sociolegal background effectively disables them from being applicable to all, even as a matter of principle. The only way to sustain the alternative conclusion would be to claim that ‘principle’, in the relevant sense, implies such dramatic estrangement from social reality as to exist only within the imagination of naive idealists.^115^ Once again, the mode of differentiation at issue here is ‘basic’ in that it goes beyond the establishment of egalitarian outcomes. The populations envisioned by sections 14 and 21 are not just *incidentally* affected but *implicitly* so—that is, by the very structure of those provisions within their proper social context. This is, in other words, an instance of *targeting*, however covert, rather than one of ‘collateral’ disadvantage.

That the 2019 Act wrongfully differentiates between the ‘dangerous others’ it implicitly targets and society at large is reflected in the concerns of several prominent commentators. For instance, Linda Logan, chair of the Magistrates Association’s Youth Court Committee, notes that her organisation is ‘particularly concerned that [the] use [of these orders] may worsen existing overrepresentation of black, Asian and minority ethnic young people in the justice system’.^116^ Similarly, the NGO Liberty has observed that sections 14 and 21 leave ‘ample scope for orders to be imposed based on considerations such as where a person lives, where they go to school, or who they are friends with; all of which may be crude proxies for race or socio-economic status’.^117^ Other bodies opposed include the Local Government Association, the Children’s Society and the All Party Parliamentary Group on Knife Crime.^118^ This collective disquiet, we believe, has at least one common conceptual root. Whether they would articulate it in these terms or not, each group mentioned above has discerned the violation of civic equality that the 2019 Act effects through the differentiation it evinces between ‘ordinary’ citizens, deserving of the protections provided by the ‘ordinary’ criminal law of England and Wales, and those ‘dangerous others’ implicitly targeted by the KCPO provisions.^119^

### D. Security and Instrumentalism

Having argued that KCPOs employ individualised control in a manner that problematically differentiates ‘dangerous others’ from the ‘virtuous majority’, it remains to be demonstrated that they instrumentalise the treatment of the former *for the benefit* of the latter. In this subsection, we argue that sections 14 and 21 of the 2019 Act do precisely that, compounding—and so cementing—their violation of civic equality. As argued above, instrumentalisation intensifies the expressive harm inflicted by differentiation and individualisation insofar as it treats those individuals subject to individualised control as a mere means to securing a majoritarian end. Such a means/ends bifurcation of erstwhile compatriots is inconsistent with their equal status and so accentuates the problematic nature of *ad hominem* criminalisation *vis-à-vis* the Rule of Law.

In the context of KCPOs, instrumentalisation operates through the manner in which the 2019 Act implicitly designates ‘both [specific] individuals and whole groups of people as potentially deviant *on grounds of risk mitigation and crime prevention*’.^120^ To this extent, justifications for the use of KCPOs, which the government pushed through as an eleventh-hour addition to the 2019 Act, are *explicitly* instrumentalist. The goal of sections 14 and 21 was ostensibly to empower police to take preventive action in situations and against individuals likely either to feature or commit knife violence, and by doing so to reduce knife crime rates in England and Wales. More general justifications have been articulated in terms of criminal law’s ‘preventive turn’, initiated in the 1990s and intensified post-9/11.^121^ Examples include the rise of regulatory criminal law and increased reliance on civil/criminal procedurally hybrid forms, notably in the context of terrorism financing and hostile environment provisions,^122^ and the forfeiture of assets arising from serious and organised crime.^123^ Such provisions are unified by their pre-emptive logic of securitisation,^124^ most often mobilised in times of uncertainty or crisis, whether actual or perceived, and both heavily cautious and risk-averse. According to this anticipatory or ‘pre-crime’ logic, punishment is no longer solely reactive or retrospective, in the mode of holding wrongdoers to account,^125^ but rather something that can be implemented prospectively for the express purpose of maximising security. In climates of elevated risk—for example, like one where knife violence in England and Wales is at a decade-long high^126^—the prospect of forestalling harms before they occur is treated as being sufficiently important to render the elevated protections of the criminal law little more than costly and untenable extravagances.^127^

Which, to bring us full circle, signals the instrumental quality of civil/criminal hybrid procedures. Indeed, KCPOs themselves are only the latest in a long line of preventive hybrids targeted towards precluding undesirable behaviour by means of such controls as those outlined earlier.^128^ Such hybrid procedures fit cleanly into the post-9/11 preventive turn and represent a broader trend of eroding criminal law’s protective elements—bypassing the presumption of innocence, characteristically sidestepping the requirement of proportionality,^129^ evading the enhanced standard of proof beyond reasonable doubt—while fortifying its intrusive elements, both in scope and reach. In their steady wearing down of due process and civil liberties, preventive hybrids like KCPOs undermine the legal protections individuals possess against state interference and control.

In normative terms, when placed under a KCPO in circumstances where no criminal conviction has been made, an individual is implicitly reduced from an equal citizen to an exploitable externality. Subject to a distinct *and additional* regime of individualised control and criminal regulation, they are, to quote Hendry: ‘both practically and symbolically … placed *outwith*. Outwith what, precisely? Outwith the (rest of) the society allegedly being protected by such securitised measures. Outwith the number of individuals aggregated to form ‘the public’, whose safety and security are apparently paramount.’^130^

Citizens who are instrumentalised in this way are no longer protected by civic equality. They are expressively alienated from the distinct mode of association established by the horizontal dimension of the Rule of Law and excluded from ‘the people’ who that law is instituted to protect. To put this in the Kantian terms employed above, those subject to regulatory tools like the KCPO exist, at least for the duration of their subjection to individualised control, outside the ‘kingdom of ends’. In the eyes of the relevant legal directives, they become mere means to support the safety of the ‘true’ body politic.

Needless to say, any society that regulates in this partial and exclusionary manner cannot claim properly to instantiate the Rule of Law, at least when the latter concept is understood in terms of civic equality. Rather than embodying that elevated mode of association, such a society instead persists as a morally impoverished polity where the equality of each citizen is not fundamental but *fungible*. Wheresoever an individual can be rendered into a means rather than an end, their equality and legal protections are liable to be sacrificed upon the alter of expediency, through the mechanisms of individualised control. This, we contend, is the *sine qua non* of *ad hominem* criminalisation and the unenviable position legislative provisions like sections 14 and 21 of the Offensive Weapons Act 2019 have placed England and Wales in today.

## 5. Conclusion

In this article, we advanced a novel theory of *ad hominem* criminalisation grounded upon a distinct egalitarian conception of the Rule of Law. Contending that societies subject to government by law are properly understood as instituting civic equality, we argued that this valuable mode of association can be undermined by particular types of governmental wrongdoing that extend beyond more traditionally understood categories, such as arbitrary punishment. Specifically, we alleged that any regulatory technique that (i) employs individualised control, (ii) explicitly or implicitly differentiates between the status of particular individuals or groups and that of society at large and (iii) instrumentalises the treatment of the former for the benefit of the latter constitutes an *ad hominem* legal regime that violates civic equality and stands in tension with the Rule of Law. Techniques of this kind represent an aberrant case of legal regulation, in fundamental opposition to the egalitarian virtues that government by law characteristically possesses.

To illustrate and exemplify this theory, we examined sections 14 and 21 of the Offensive Weapons Act 2019, which establish the availability of controversial Knife Crime Prevention Orders. These orders, infamously ‘introduced without proper consultation or a firm evidence base to rely on’,^131^ establish a civil/criminal hybrid regime that is paradigmatic of the *ad hominem* criminalisation that concerned us here. With a ‘two-step process’^132^ of individualised control that fast-tracks those subject to them into becoming punishable by the criminal law *without* undertaking criminal activity, KCPOs place a heavy burden upon the state to justify the regulatory asymmetry they create between those subject to them and those individuals who remain regulated by the ‘ordinary’ criminal law. Unfortunately, given the unjust race-, age- and class-based differentiations that mar the social backdrop to the relevant legislative provisions,^133^ no such justification can be mounted. KCPOs both depart from and exacerbate the extant social prejudices that exist within England and Wales, creating, in effect, a ‘second class’ of citizen that exists outwith the ‘virtuous majority’ that those orders seek to protect. This ‘dangerous other’—who is characteristically young, Black, male and poor—is then *further* alienated by being instrumentalised as a means to secure the safety of the public at large.

In practical terms, this problematic trifecta of individualised control, wrongful differentiation and exploitative instrumentalisation divests those subject to KCPOs of important procedural rights and relegates them to a procedurally hybrid fast track towards criminal punishment. In expressive terms, it effects a threefold alienation of such individuals from full membership within society, denies them civic equality as subjects of the law and violates the precept that the ordinary criminal law should, in principle, be applicable to all. Taken together, these factors place the issuing of KCPOs in fundamental tension with the horizontal dimension of legality: that distinct mode of association whereby government under law transforms a society into a community of equals.

## Footnotes

1 eg TRS Allan, *Constitutional Justice: A Liberal Theory of the Rule of Law* (OUP 2001) 4, 233.

2 ibid.

3 (1996) 138 ALR 577.

4 Allan (n 1) 238.

5 ibid 237.

6 ibid.

7 WB Gallie, ‘Essentially Contested Concepts’ (1955) 56 Proceedings of the Aristotelian Society 167.

8 Gerald Postema, ‘Fidelity in Law’s Commonwealth’ in Lisa M Austin and Dennis Klimchuck (eds), *Private Law and the Rule of Law* (OUP 2014) 19–20.

9 Ronald Dworkin, *Law’s Empire* (Fontana Press 1986) 93.

10 Lon Fuller, *The Morality of Law* (Yale UP 1969) 130.

11 John Finnis, *Natural Law and Natural Rights* (OUP 2011) 270–6.

12 Friedrich Hayek, *The Road to Serfdom* (Routledge Press 1944) 54.

13 Neil MacCormick, *Rhetoric and the Rule of Law: A Theory of Legal Reasoning* (OUP 2005) 12.

14 Joseph Raz, *The Authority of Law* (OUP 1979) 212.

15 Jeremy Waldron, ‘The Concept and the Rule of Law’ (2008) 43 Ga L Rev 1, 6.

16 Raz (n 14) 219–20.

17 Tom Bingham, *The Rule of Law* (Penguin Books 2010) 66–8.

18 Fuller (n 10) 46–91.

19 Raz (n 14) 220.

20 Postema (n 8) 17.

21 Dworkin (n 9) 200, 213–14.

22 Lon Fuller, ‘Postivism and Fidelity to Law—A Reply to Professor Hart’ (1957) 71 Harv L Rev 630.

23 Postema (n 8) 21.

24 Hannah Arendt, *The Origins of Totalitarianism* (Meridian Books 1958) 301.

25 It is immaterial for present purposes whether this basic equality is ‘natural’ in the sense that the rule of law simply recognises the fundamental equality of all human beings, or whether it is constructed, in the sense of arising from the act of political association. For more on these two ways of understanding basic equality, see Jeremy Waldron, *One Another’s Equals. The Basis of Human Equality* (Belknap Press 2017) 50–61.

26 Jennifer Hendry, ‘“The Usual Suspects”: Knife Crime Prevention Orders & the “Difficult” Regulatory Subject’ (2021) Brit J Criminol <https://doi.org/10.1093/bjc/azab063>.

27 RA Duff, *Answering for Crime* (Hart Publishing 2009) 43–56; Jo Shaw, *The People in Question: Citizens and Constitutions in Uncertain Times* (Bristol UP 2020) 79, 155.

28 Thomas Nagel, ‘Personal Rights and Public Space’ (1995) 24(2) Philosophy & Public Affairs 83, 85.

29 Waldron (n 25) 49.

30 Dworkin (n 9) 189–90.

31 Postema (n 8) 25.

32 ibid 37–8.

33 Jean-Jacques Rousseau, ‘On the Social Contract, or Principles of Political Right’ in *The Basic Political Writings* (Donald A Cress tr, Hackett 1987) 150: ‘For since the sovereign is formed entirely from the private individuals who make it up, it neither has nor could have an interest contrary to theirs.’

34 RA Duff, ‘Inclusion and Exclusion: Citizen, Subjects and Outlaws’ (1998) 51 CLP 241, 253–6.

35 Rousseau (n 33) 157: ‘Thus, just as a private will cannot represent the general will, the general will, for its part, alters its nature when it has a particular object; and as general, it is unable to render a decision on either a man or a state of affairs.’

36 As we detail below, such problematic differentiation must be more than purely formal. It is plausible, for example, that *some* individualised criminal regulation might be consistent with civic equality. Under such circumstances, any formal violation of the requirement that laws be general is *prima facie* only. As we define that phenomenon, *ad hominem* criminalisation can make no such claim, since it necessarily differentiates between regulatory subjects at the substantive level as well.

37 Finnis (n 11) 9–18.

38 Fuller (n 10) 46–9.

39 Immanuel Kant, *The Metaphysics of Morals* (Mary Gregor tr, CUP 2017) 49 [6:255–7].

40 Thomas Hobbes, *Leviathan* (JCA Gaskin ed, OUP 1996) 122.

41 Allan (n 1) 38–40.

42 See also Habermas, who argues, ‘Dworkin’s basic standard of “integrity” stands for the political ideal by which citizens recognize one another as free and equal, and are obligated to realize the basic norm of equal concern and respect for each person in the practices of society’: Jürgen Habermas, *Between Facts and Norms: Contributions to a Discourse Theory of Law and Democracy* (Polity Press 1997) 215.

43 Dworkin (n 9) 189–90.

44 Allan (n 1) 24–5.

45 Kant (n 39) 99 [6:314]; Friedrich Hayek, *The Constitution of Liberty* (Chicago UP 1960) 153.

46 Dworkin (n 9) 189; Postema (n 4) 29; Duff (n 34) 255.

47 AV Dicey, *Introduction to the Study of The Law of the Constitution* (first published 1885, 10th edn, Macmillan 1959) 193.

48 Raz (n 14) 215–16.

49 On the distinction between force and coercion, see Robert Nozick, ‘Coercion’ in Sidney Morgen Besser, Patrick Souppes and Morton White (eds), *Philosophy, Science, and Method: Essays in Honour of Ernest Nagel* (St Martin’s Press 1969).

50 Daniel Halliday, *Inheritance of Wealth: Justice, Equality, and the Right to Bequeath* (OUP 2018) 102–54.

51 Aristotle, *Nicomachean Ethics*, V.3. 1131a10–b15; *Politics*, III.9.1280 a8–15, III. 12. 1282b18–23.

52 Martha Fineman, ‘The Vulnerable Subject: Anchoring Equality in the Human Condition’ (2008) 20(1) Yale J L & Feminism 1, 2–5.

53 Sandra Fredman, ‘Substantive Equality Revisited’ (2016) 14 ICON 712.

54 On these various conceptions of equality, see Amartya Sen, ‘Equality of What?’ in S McMurrin (ed), *Tanner Lectures on Human Values, I* (CUP 1980); Gerry Cohen, ‘Equality of What? on Welfare, Goods, and Capabilities’ in Martha Nussbaum and Amartya Sen (eds), *The Quality of Life* (OUP 1993).

55 Alex Green, ‘Three Models of Political Membership: Delineating ‘The People in Question” (2021) 41 OJLS 565, 568–76.

56 This is not to suggest that *all* such violations of civic equality necessarily constitute *ad hominem* criminalisation. As we argue below, the latter phenomenon constitutes a uniquely complex and egregious wrong of this broader type.

57 RA Duff, ‘Perversions and Subversions of Criminal Law’ in RA Duff and others (eds), *The Boundaries of Criminal Law* (OUP 2010) 89.

58 Ernest J Weinrib, *The Idea of Private Law* (OUP 2012) 133–42.

59 RA Duff, ‘A Criminal Law to Call Our Own?’ (2017) 111 Northwestern University Law Review 1491, 1495.

60 Weinrib (n 58) 105–7.

61 For an historical account of the denial of citizenship to and social exclusion of perceived ‘deviants’ on moral grounds, see David Garland, *Punishment and Welfare: A History of Penal Strategies* (Gower 1985).

62 Michel Foucault, *Discipline and Punish: The Birth of the Prison* (Penguin Books 1991) 90.

63 Whilst this argument focuses upon physical liberty, it nonetheless coheres with various non-physical restrictions of civic entitlement, such as the suspension of voting rights. Indeed, it arguably supports our account of criminal sanctions that such civic restrictions characteristically coincide with incarceration: the civic exclusion effected by incarceration is reinforced where incarcerated individuals are also disenfranchised for the duration. Moreover, if restrictions upon physical liberty are understood not as just *one* mode through which criminal justice is implemented but rather as *the central case* of criminal punishment, then the expressive force of *other* non-physical sanctions, such as fines, can be explained by implication. If the central case of criminal punishment is one in which convicted persons are socially excluded, then the mere designation of an offence as criminal will characteristically insinuate severe transgression: Victor Tadros, ‘Criminalization and Regulation’ in Duff and others (n 57) 164–5. This establishes one way in which criminal fines can be distinguished from orders for civil compensation. See generally Pat O’Malley, ‘Theorizing Fines’ (2009) 11 Punishment and Society 67.

64 Richard Dagger, ‘Republicanism and Crime’ in Samantha Besson and José Luis Martí (eds), *Legal Republicanism: National and International Perspectives* (OUP 2009) 148; cf Andrew Ashworth and Lucia Zedner, ‘Just Prevention: Preventative Rationales and the Limits of the Criminal Law’ in RA Duff and Stuart Green (eds), *Philosophical Foundations of Criminal Law* (OUP 2011) 282.

65 As such, our focus is ultimately upon the *content* of the criminal law—that is, which and how many norms it contains—rather than upon the *process* by which it is enforced. Therefore, although we exemplify our argument with reference to a regulatory technique that takes a very particular procedural form (that is, the KCPO), our interest in procedure extends only so far as the latter informs the substantive concerns we raise in relation to *ad hominem* criminalisation. To that extent, our argument differs in focus from Ericson’s theory of ‘counter-law’, which primarily examines those norms that undermine the procedural guarantees that criminal law traditionally provides: Richard Ericson, *Crime in an Insecure World* (Polity Press 2007) 207. Moreover, although Ericson notes how antisocial behaviour laws permit discretionary threat assessments that risk those subject to them being ‘exclu[ded] from membership in a valued community’ (ibid 200, 211), he does not connect this critique to the idea of civic equality, which forms our analytical focus here.

66 Ronald Dworkin, ‘Hart’s Postscript and the Character of Political Philosophy’ (2004) 24 OJLS 1, 29 (emphasis added).

67 See generally Christian List and Philip Pettit, *Group Agency: The Possibility, Design, and Status of Corporate Agents* (OUP 2011).

68 Duff (n 34) 245–6.

69 Liam Murphy, *What Makes Law: An Introduction to the Philosophy of Law* (CUP 2015) 137–9.

70 That governments are sometimes institutionally accountable does not remove the need for this trust. See Melaine Ehren, Andrew Paterson and Jacqueline Baxter, ‘Accountability and Trust: Two Sides of the Same Coin?’ (2020) 21 Journal of Educational Change 183.

71 When using such terms, our intention is not to examine, as Ramsay does, the coherence of an (allegedly) representative government claiming legitimate authority to protect ‘the public’ against ‘those who manifest a disposition that fails to reassure, [and] those who are perceived as dangerous’: Peter Ramsay, *The Insecurity State: Vulnerable Autonomy and the Right to Security in the Criminal Law* (OUP 2012) 65. Instead, we argue that any such bifurcation of society is normatively suspect *vis-à-vis* the Rule of Law, whatever the position might be in relation to representative government. Moreover, unlike Ramsay, our analytical focus is *not* upon the existence (or otherwise) of a legal infrastructure that purportedly grounds a general ‘right to security’, which he describes in places as a right to *feel* secure (ibid 2–6). Instead, we focus upon the normative permissibility of a particular regulatory technique (that is, *ad hominem* criminalisation) from the perspective of status-egalitarianism.

72 Ramsay identifies this instrumentalisation in relation to the now-defunct ASBO: ibid 59–61, 65, 79.

73 Allan (n 1) 246 (citing *Hurtado v California* 110 US 516 (1884), 535–6).

74 ibid 39.

75 Adam Crawford, ‘Governing through Anti-Social Behaviour: Regulatory Challenges to Criminal Justice’ (2009) 49 Brit J Criminol 810, 820.

76 Allan (n 1) 238.

77 Finnis (n 11) 154 (emphasis added).

78 [1967] 1 AC 259.

79 To this extent, *ad hominem* criminalisation is a more discrete phenomenon than what Lacey refers to as ‘status criminalisation’. See Nicola Lacey, *In Search of Criminal Responsibility: Ideas, Interests, and Institutions* (OUP 2016) 158.

80 On ‘enemy criminal law’, see Daniel Ohana, ‘Trust, Distrust and Reassurance: Diversion and Preventive Orders through the Prism of Feindstrafrecht’ (2010) 73 MLR 721; Daniel Ohana, ‘Günter Jakob’s *Feindstrafretch*: A Dispassionate Account’ in Markus Dubber (ed), *Foundational Texts in Modern Criminal Law* (OUP 2014).

81 Carl Schmitt, *The Concept of the Political* (G Schwab tr, Chicago UP 2007) 26–9, 38, 43–4.

82 Henrique Carvalho, *The Preventive Turn in Criminal Law* (OUP 2017) 182.

83 Hendry (n 26).

84 This distinction between the complete set of criminal laws that govern ‘ordinary’ or ‘virtuous’ citizens and the larger, more restrictive set that governs ‘dangerous others’ is not to be confused with the distinction that Dubber makes between criminal law as such and ‘criminal police’. For Dubber, policing constitutes an altogether different mode of governance from that of the criminal law. By contrast, our concern is rather that morally problematic differentiation, when attached to individualised control, constitutes an unjust bifurcation *within* the criminal law itself. See Markus Dubber, ‘Criminal Police and Criminal Law in the Rechsstat’ in Markus Dubber and Mariana Valverde (eds), *Police and the Liberal State* (Stanford UP 2008) 95–6; Markus Dubber, *The Police Power: Patriarchy and the Foundations of American Government* (Columbia UP 2005) 3.

85 This diagnosis is therefore ‘deeper’ than concluding that the egalitarian merits of a particular law are *qualified*. Laws that differentiate *vis-à-vis* basic status do more than produce inegalitarian outcomes: they *construct* status-inequality by *deconstructing* the legal expression(s) of civic membership.

86 Dworkin (n 9) 213.

87 Finnis (n 11) 154.

88 Sen (n 54).

89 Ramsay (n 71) 120.

90 Immanuel Kant, *Groundwork of the Metaphysics of Morals* (Mary Gregor tr, CUP 1998) 41.

91 Home Office, Guidance on Knife Crime Prevention Orders (Home Office 2019).

92 Hendry (n 26).

93 The ASBO was available between 1998 and 2014 in England and Wales to anyone aged 10 or over. ASBOs were imposed by courts employing the criminal standard of proof and stipulated only negative requirements. This form of regulation has a long history of being treated as suspect *vis-à-vis* the Rule of Law. See, eg Andrew Ashworth and others, ‘Neighbouring on the Oppressive: The Government’s “Anti-Social Behaviour Order” Proposals (1998) 16 Criminal Justice 7.

94 See generally Stephen Shute, ‘Rationalising Civil Preventive Orders: Opportunities for Reform’ in JJ Child and RA Duff (eds), *Criminal Law Reform Now: Proposals and Critique* (Hart Publishing 2018); Carvalho (n 82); A Ashworth and L Zedner, *Preventive Justice* (OUP 2014).

95 As such, although our discussion of KCPOs covers some thematically similar ground, it is methodologically distinct from other work which addresses preventive regulation. Ramsay, for example, offers an ‘immanent critique’ of the right to security as an emergent legal phenomenon (Ramsay (n 71) 4, 9), which proceeds in a heuristic and therefore explanatory manner, turning only to normative critique insofar as it helps to identify internal inconsistencies and so ‘lay bare [security’s] conceptual and normative structure’ within the object of interpretation. By contrast, we provide an *external* normative critique of (i) *ad hominem* criminalisation conceived as a moral/political wrong and (ii) one preventive regulatory technique (that is, the KCPO), the latter of which is employed primarily to facilitate the former. Since this critique departs from a robustly egalitarian external perspective, it operates in sharp contrast to Ramsay’s primarily explanatory undertaking.

96 KCPOs can also be made on conviction under section 19 of the same Act, where the prosecution applies for one in addition to either: (i) the sentence imposed in respect of the offence (7a); or (ii) a conditional discharge order (7b). However, while, in terms of due process considerations, this appears to be preferable, the evidence admissible for determining the suitability of such an order is not restricted by the usual requirements for criminal proceedings (subsection 9). (The 2019 published Home Office Guidance not only states explicitly that the strict rules of criminal evidence do not apply in these circumstances, but also that the application can ‘rely on the facts of the case for which the defendant has been convicted’: Home Office (n 91) 6.) Although this arguably gives rise to separate procedural concerns, these are not our focus.

97 Offensive Weapons Act 2019, s 29(2).

98 Hendry (n 26) 4.

99 For an analogous point in relation to ASBOs, see JC Smith, ‘Antisocial Behaviour Order: Whether Proceedings Civil or Criminal in Nature’ [2003] Crim LR 269, 271.

100 Lindsay Farmer, *Making the Modern Criminal Law* (OUP 2016) 188–92.

101 Hannah Quirk, Toby Seddon and Graham Smith, ‘Regulation & Criminal Justice: Exploring the Connections and Disconnections’ in H Quirk, T Seddon and G Smith (eds), *Regulation & Criminal Justice: Developing a New Framework for Research & Policy Development* (CUP 2010) 20.

102 Crawford (n 75).

103 By creating this asymmetry, individualisation distinguishes, in both procedural and normative terms, the KCPO from the *crime* of knife possession: see Prevention of Crime Act 1953, s 1(1); Criminal Justice Act 1988, ss 139(1) and 139A(1). Although such offences are often policed in a manner that betrays problematic differentiation (see below), they do not legally *individuate* in the same manner. They are, to that extent, ‘ordinary’ or ‘standard’ criminal offences and therefore do not pose the civic (in)egalitarian problem of rendering *some* individuals subject to norms that their compatriots are not. For more on the *distinctive* aspects of crimes of possession, see Markus Dubber, ‘The Possession Paradigm: The Special Part and the Police Power Model of Criminal Process’ in RA Duff and Stuart Green (eds), *Defining Crimes: Essays on the Special Part of the Criminal Law* (OUP 2005).

104 Andrew Simester and Andreas von Hirsch, ‘Two-Step Prohibitions’ in Andreas von Hirsch and Andrew Simester (eds), *Incivilities: Regulating Offensive Behaviour* (OUP 2006) 181–2.

105 John Braithwaite, *Restorative Justice & Responsive Regulation* (2002 OUP) 30.

106 Hendry (n 26) 9.

107 Home Office (n 91) 18.

108 HL Deb 26 February 2019, vol 796, col 191.

109 Hendry (n 26) 6; see also D Garland, *The Culture of Control* (OUP 2001) 136; P Williams and B Clarke, ‘The Black Criminal Other as an Object of Social Control’ (2018) 7 Social Sciences 234.

110 HL (n 108) col 195.

111 ibid col 196.

112 See generally Carrie Menkel-Meadow, ‘Uses and Abuses of Socio-Legal Theory’ in Naomi Creutzfeldt, Marc Mason and Kirsten McConnachie (eds), *Routledge Handbook of Socio-Legal Theory and Methods* (Routledge 2019) 35–57; Roger Cotterrell, ‘Subverting Orthodoxy, Making Law Central: A View of Sociolegal Studies’ (2002) 29 Journal of Law and Society 632.

113 Standing Committee for Youth Justice, ‘SCYJ Submission to Consultation on Knife Crime Prevention Orders Guidance’, 3 <http://scyj.org.uk/wp-content/uploads/2019/09/FINAL-SCYJ-Submission-to-Consultation-on-Knife-Crime-Prevention-Orders-Guidance.pdf>.

114 ibid.

115 See generally Nicola Lacey (2009) ‘Historicising Criminalisation: Conceptual and Empirical Issues’, (2009) 72(6) MLR 936.

116 Linda Logan (6 March 2020) <www.magistrates-association.org.uk/News-and-Comments/knife-crime-prevention-orders-to-be-piloted-in-london>.

117 R Comyn, ‘Knife Crime Prevention Orders Would Only Harm the Fight against Violent Crime’ ( 6 February 2019) <www.libertyhumanrights.org.uk/issue/knife-crime-prevention-orders-would-only-harm-the-fight-against-violent-crime/>.

118 HL (n 108) col 200.

119 Ohana (n 80).

120 Hendry (n 26) (original emphasis). See also Ericson (n 65) 206–8, 213–14.

121 See generally Carvalho (n 82); Ashworth and Zedner (n 94).

122 Lucia Zedner, ‘The Hostile Border: Crimmigration, Counter-Terrorism, or Crossing the Line on Rights?’ (2019) 22 New Crim L Rev 318; Jennifer Hendry, ‘The Hostile Environment and Crimmigration: Blurring the Lines between Civil and Criminal Law’ (2020) 76 Soundings 26; see generally Colin King, Clive Walker and Jimmy Gurulé (eds), *The Palgrave Handbook of Criminal and Terrorism Financing Law* (Palgrave Macmillan 2018).

123 Colin King and Jennifer Hendry, *The Civil Recovery of Criminal Assets* (OUP forthcoming); Jennifer Hendry and Colin King, ‘Expediency, Legitimacy, and the Rule of Law: A Systems Perspective on Civil/Criminal Procedural Hybrids’ (2017) 11 Criminal Law & Philosophy 733.

124 Lucia Zedner, *Security* (Routledge 2009) 73.

125 Victor Tadros, *Criminal Responsibility* (OUP 2005) 76–98.

126
*Office for National Statistics*, data for year ending December 2019 <www.ons.gov.uk/peoplepopulationandcommunity/crimeandjustice/datasets/policeforceareadatatables> accessed 20 June 2020.

127 Zedner (n 124) 80; Michael Ignatieff, *The Lesser Evil: Political Ethics in an Age of Terror* (Edinburgh UP 2004); Andreas Ashworth, ‘Four Threats to the Presumption of Innocence’ (2006) 123 SALJ 62.

128 David Garland, *The Culture of Control* (OUP 2001).

129 It should be noted that there are proportionality limits on civil recovery orders; see King and Hendry (n 123).

130 Hendry (n 26) 13. Emphasis in the original.

131 Logan (n 116).

132 Andrew Simester and Andreas von Hirsch, ‘Regulating Offensive Conduct through Two-Step Prohibitions’ in von Hirsch and Simester, *Incivilities* (n 104) 173–217.

133 Notably both race and age—alongside disability, gender reassignment, sex, sexual orientation, marriage and civil partnership, pregnancy and maternity, and religion or belief—are protected characteristics under the Equality Act 2010. This observation further supports our assertion that broader sociolegal considerations of context and background conditions are vital to see beyond mere formal equality.

## Notes

Dr Green’s work on this article was supported by a *Modern Law Review* Covid-19 Response Fellowship.

Dr Hendry’s work on this article was supported by an Arts and Humanities Research Council (AHRC) Leadership Fellowship award (Early Career) 2019–21 (grant number AH/S006680/1). The authors are grateful to Chloë Kennedy, Colin King, Findlay Stark, Neil Walker and Simon NM Young for their comments on an earlier draft. Thanks are also due to two anonymous reviewers and to the participants of the Edinburgh–Glasgow Virtual Criminal Law Seminar Series. The usual disclaimer applies.

